# Structural Investigation of Betulinic Acid Plasma Metabolites by Tandem Mass Spectrometry

**DOI:** 10.3390/molecules27217359

**Published:** 2022-10-29

**Authors:** Roxana Ghiulai, Marius Mioc, Roxana Racoviceanu, Alexandra Prodea, Andreea Milan, Dorina Coricovac, Cristina Dehelean, Ştefana Avram, Alina D. Zamfir, Cristian V. A. Munteanu, Viviana Ivan, Codruța Şoica

**Affiliations:** 1 Department of Pharmacology-Pharmacotherapy, Victor Babes University of Medicine and Pharmacy Timisoara, 2nd Eftimie Murgu Sq., 300041 Timisoara, Romania; 2 Research Centre for Pharmaco-Toxicological Evaluation, Victor Babes University of Medicine and Pharmacy Timisoara, 2nd Eftimie Murgu Sq., 300041 Timisoara, Romania; 3 Department of Pharmaceutical Chemistry, Faculty of Pharmacy, Victor Babes University of Medicine and Pharmacy Timisoara, 2nd Eftimie Murgu Sq., 300041 Timisoara, Romania; 4 Department of Toxicology, Faculty of Pharmacy, Victor Babes University of Medicine and Pharmacy Timisoara, 2nd Eftimie Murgu Sq., 300041 Timisoara, Romania; 5 Department of Pharmacognosy, Faculty of Pharmacy, Victor Babes University of Medicine and Pharmacy Timisoara, 2nd Eftimie Murgu Sq., 300041 Timisoara, Romania; 6 Department of Condensed Matter, National Institute for Research and Development in Electrochemistry and Condensed Matter, 300224 Timisoara, Romania; 7 Faculty of Physics, West University of Timisoara, 300223 Timisoara, Romania; 8 Institute of Biochemistry of the Romanian Academy, Splaiul Independent, ei 296, 060031 Bucharest, Romania; 9 Department of Internal Medicine II, Faculty of Medicine, Victor Babes University of Medicine and Pharmacy Timisoara, 2nd Eftimie Murgu S.2, 300041 Timisoara, Romania

**Keywords:** betulinic acid, phase I metabolites, phase II metabolites, ESI CID MS/MS

## Abstract

Betulinic acid (BA) has been extensively studied in recent years mainly for its antiproliferative and antitumor effect in various types of cancers. Limited data are available regarding the pharmacokinetic profile of BA, particularly its metabolic transformation in vivo. In this study, we present the screening and structural investigations by ESI Orbitrap MS in the negative ion mode and CID MS/MS of phase I and phase II metabolites detected in mouse plasma after the intraperitoneal administration of a nanoemulsion containing BA in SKH 1 female mice. Obtained results indicate that the main phase I metabolic reactions that BA undergoes are monohydroxylation, dihydroxylation, oxidation and hydrogenation, while phase II reactions involved sulfation, glucuronidation and methylation. The fragmentation pathway for BA and its plasma metabolites were elucidated by sequencing of the precursor ions by CID MS MS experiments.

## 1. Introduction

**Figure 1:**
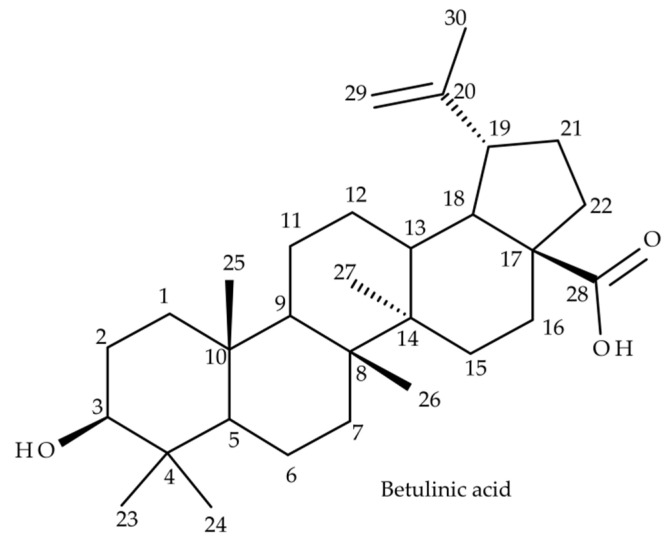
Chemical structure of betulinic acid (BA) (3β, hydroxy-lup-20(29)-en-28-oic acid).

Betulinic acid (BA) (3β, hydroxy-lup-20(29)-en-28-oic acid) (Figure 1) is a lupane-skeleton pentacyclic triterpene secondary plant metabolite. BA is widely distributed in natural occurring sources, considerable amounts being found throughout Betulaceae species, in the outer layers of birch bark [1]. Some vegetal sources with high amounts of BA are an important part of natural remedies that include traditional Chinese medicine such as Ziziphi Spinosae semen (Rhamnaceae) [2] or Indian medicinal remedies such as *Nyctanthes arbor*-*tristis* [3]. In recent decades, BA has been extensively investigated during in vitro and in vivo studies that demonstrated a wide range of biological and pharmacological activities, the most high profile being its anticancer potential, already recognized by the National Cancer Institute of the USA [4]. BA displays well documented cytotoxicity activities mainly by triggering the mitochondrial pathway of apoptosis in cancer cells [5] exerted on various types of cancers such as melanoma [6], colorectal cancer [7], ovarian cancer [8], cervical cancer [9] or breast cancer [10]. Hence BA has an immense potential of becoming an antineoplastic agent that combines high anticancer activity, low overall toxicity and high selectivity. In addition, BA has demonstrated other biological effects as well, such as a very potent anti-inflammatory activity [11] and antiviral effects exerted on HIV, HSV and hepatitis B [12]. Not least, BA has displayed antidiabetic activity in type 2 diabetes, improved the outcome of metabolic syndrome [13] and was demonstrated to promote wound healing [14]. One major drawback of BA is represented by its low water solubility which negatively affects its oral bioavailability, thus limiting its pharmacological activity. To overcome these shortcomings and to improve its pharmacokinetic features, several strategies have been employed such as cyclodextrin complexation [15] and several nanoformulations containing BA, such as liposomes, nanoemulsions, nanoparticles or carbon nanotubes [16]. In drug development, a mandatory stage lies in disclosing its complete pharmacokinetic parameters, such as absorption, distribution, metabolism and excretion; briefly, its ADME profile [17]. Drug metabolism represents a very complex process that occurs in the liver, during which lipophilic molecules undergo metabolic transformations to enhance their hydrosolubility and to enable their renal excretion. Generally, drug biotransformation consists in two successive stages represented by phase I and phase II metabolic reactions. The end result of these reactions is mainly the inactivation and detoxification of xenobiotics in the liver [18]. However in some cases, drug metabolism leads to compounds that possess enhanced pharmacological activity mainly by a superior bioavailability such as the case of well-known medicinal drugs, isosorbide mononitrate, ambroxol or nortriptyline [19]. Despite the remarkable results in disclosing the underlying molecular mechanisms for most of BA’s biological and pharmacological activities during in vitro and in vivo studies, there is limited information regarding its in vivo ADME profile. Among the premier studies that disclosed some pharmacokinetic parameters was one conducted by Udeani et al. [20], during which the tissue distribution and half-life (T_1/2_) of BA-polyvinylpyrrolidone (PVP) complex after intraperitoneal (IP) administration in CD-1 mice was elucidated. In addition, Cheng et al. [21] investigated the extent of binding of BA to plasma proteins after IP administration in mouse and rats. The available data regarding the in vivo metabolism of BA are even more limited, hence only a couple of studies have been published. Recently, Zhang et al. [22] reported on some phase I metabolites identified in the feces of Sprague-Dawley rats after oral administration of BA-carboxyl methyl cellulose solution. Another study conducted by Li et al. [23] reported on other phase I and phase II metabolites of BA mainly identified in the feces of Sprague-Dawley rats after oral administration of BA, spinosin, isovitexin and jujuboside together with Ziziphi Spinosae semen extract. Under these circumstances, more studies are needed to enrich and complete the in vivo metabolic profile of BA. Currently, mass spectrometry (MS)-based analytical techniques such as high resolution MS (HRMS), LC-MS, GC-MS and NMR analysis are state-of-the-art analytical techniques applied in metabolomics to identify and to elucidate the chemical structure of these small molecules [24]. HRMS techniques are widely used for the metabolomics of both endogenous [25] and xenobiotics due to their high sensitivity and accuracy. Among the preferred ionization techniques, electrospray ionization (ESI) and APCI combined with high resolution analyzers such as Q-TOF or Orbitrap are the most frequent, while structural investigations are performed through tandem MS or MS/MS experiments by collision-induced dissociation (CID) or HCD [26]. The current study is focused on the identification and structural characterization of both phase I and phase II metabolites of BA detected in SKH1 mouse plasma. The analytical platform for all the resulted metabolomic data consisted in ESI Orbitrap MS in the negative ion mode and CID MS/MS. The obtained results deliver new information regarding the in vivo metabolic pathway of BA and document the fragmentation pathway of BA and its metabolites. All combined, the current study contributes to elucidate the metabolomic profile of BA and can represent a support for future studies.

## 2. Results and Discussion

### 2.1. Screening of BA and BA Metabolites by Negative Ion Mode ESI Orbitrap MS in Plasma Samples

Aliquots of 10 µL of deproteinized plasma samples were loaded into the LTQ Orbitrap Velos Pro^TM^ mass spectrometer for negative ion mode screening experiments. Screening mass spectra were recorded in 100–1000 mass range. Betulinic acid was identified as the deprotonated ion [M-H]^−^ at *m*/*z* 455.35. MS screening experiments enabled the identification of 13 phase I and phase II metabolic products of BA. The main phase I metabolic reactions BA underwent were oxidation, monohydroxylation, dihydroxylation and hydrogenation, while the major metabolic pathways during phase II reaction were sulfation, glucuronidation and methylation. The proposed metabolic products and their molecular modifications are depicted in Table 1.

#### 2.1.1. Phase I Metabolic Products

Phase I metabolism of natural phytocompounds, as for medicinal drugs, aims to convert liposoluble molecules into more polar ones by introducing or liberating a polar substituent. The resulting metabolites may have their biological activity both altered or unchanged and are the outcome of complex metabolic reactions such as oxidation, reduction and hydrolysis catalyzed by oxidases, reductases and esterases [27]. Oxidation reactions are mostly dependent and mediated by the isoforms of cytochrome P450, out of which CYP2C, CYP2D6 and CYP3A are responsible for the metabolic fate of nearly 75% of drugs. They are present in the endoplasmic reticulum of hepatocytes and intestines, involve a large variety of reactions such as hydroxylations, epoxidations or oxidative dealkylations, and exert by the addition of one or more oxygen atoms to the structure of the parent drug [28]. Since BA is a highly hydrophobic molecule, it is expected to undergo phase I metabolic changes as well.

The screening of plasma samples revealed the presence of the [M-H]^−^ ion at *m*/*z* 457.37 (**M1**), which has a molecular weight (Mw) 2 Da more than BA, corresponding to the addition of 2 H atoms and was assigned according to the mass calculations to the product of a hydrogenation reaction by double bond reduction occurring at the isopropenyl group (C_20_-C_29_) of ring E. Hydrogenation is a common metabolic reaction reported both for human steroid metabolism and for the metabolism of phytochemicals that bear double bonds in their structure, such as flavonoids [29], terpenes [30] or phenylpropanoid glycosides [31]. Next, the precursor ion identified as [M-H]^−^ at *m*/*z* 471.35 (**M2** ) has a Mw 16 Da more than BA, corresponding to the addition of an O atom as a result of a metabolic reaction that modified most likely one of the methyl substituents to hydroxymethyl, as reported both for medicinal drugs [32] and phytocompounds [23]. Hence, *m*/*z* 471.35 was assigned as the monohydroxylated metabolite of BA. The purpose of hydroxylation, an oxidative metabolic reaction, is to increase the hydrosolubility of less polar compounds, in most cases generating active compounds. Moreover, monohydroxylated metabolites were already reported as in vivo metabolic products for asiatic and madecassic acid [33], maslinic acid [34] and betulinic acid [23]. The precursor ion identified as [M-H]^−^ at *m*/*z* 485.33 (**M3**) has a Mw 30 Da more than BA, corresponding to the addition of 2 O atoms while eliminating 2 H atoms. This second step of the oxidation reaction most likely altered the already available hydroxymethyl substituent; the end result of this two-step oxidation is the conversion of the methyl substituent to carboxyl, generating an inactive metabolite, as previously reported [23,32]. Furthermore, the MS screening spectrum indicated also the formation of the precursor ion at *m*/*z* 487.34 (**M4**), which has a Mw 32 Da more than BA, corresponding to the addition of two O atoms as a result of a two-site hydroxylation metabolic reaction, most likely converting two methyl substituents to hydroxymethyl ones. A dihydroxylation metabolic reaction was previously reported by other studies that investigated the in vivo or in vitro metabolism of: (i) triterpenes, such as maslinic acid [34], asiatic and madecassic acid [33], betulinic acid [35], (ii) diterpenes [36] and (iii) alkaloids [37].

#### 2.1.2. Phase II Metabolic Products

Phase II metabolism consists in conjugation reactions such as glucuronidation, sulfation, acetylation, methylation and glycine conjugation or glutathione conjugation of the parent drug or the already formed phase I metabolites with endogenous substrates such as UDP glucuronic acid (GluA), phosphoadenosyl phosphosulfate, Acetyl-CoA, gluthatione, glycine or S-adenosyl-methionine, catalyzed by specific transferases [38]. Glucuronidation and/or sulfation are the most common types of metabolism for phytocompounds such as flavonoids, terpenoids or alkaloids [27]. The resulting metabolic products are generally inactive, more polar and readier to undergo renal or biliary excretion.

The screening mass spectra of analyzed samples indicated the presence of the precursor ion identified as [M-H]^−^ at *m*/*z* 469.37 (**M5**),which has a Mw 14 Da more than BA, and was assigned according to the mass calculations to the methylated metabolite of BA, which is consistent with previous findings that reported methylated metabolites for echinocystic acid, a triterpene [39], as well as for BA [22,23]. Although methylation is a minor conjugation reaction for xenobiotic transformation, being more common for endogenous neurotransmitters, it still remains a metabolic pathway for a large number of medicinal drugs and phytocompounds [38]. It is catalyzed by methytransferases and the resulting metabolic products are either active or inactive, while their solubility is almost the same as that of the parent drug. Since BA possesses a hydroxyl substituent, most probably the parent ion underwent O-methylation.

In addition, the [M-H]^−^ ion identified at *m*/*z* 535.31 (**M6**) has a Mw 80 Da more than BA and was assigned according to the mass calculations to the sulfoconjugate metabolite of BA. Sulfoconjugation is one of the most important phase II metabolic reactions affecting a great variety of compounds such as hormones, bile acids, medicinal drugs and various classes of phytocompounds, including triterpenes. Addition of the sulfonate group occurs both directly to the parent compound or to its phase I metabolite, leading to the formation of a non-toxic water–soluble compound ready for excretion [38]. The reaction is catalyzed by sulfotransferases (SULTs) that are capable to transfer (SO_3_) from 3′–phosphoadenosine 5′–phosphosulfate (PAPS) to hydroxyl or amino groups [40]. Hence, in the case of BA, the reaction most likely occurred at the –OH substituent present on ring A. These findings are consistent with the data of Li et al. [23] on the in vivo metabolism of BA, the sulfoconjugate being one of the reported metabolites. In addition, the result of sulfoconjugation after in vivo administration in rats was also reported for betulin [41].

Next, the [M-H^+^]^−^ ion detected at *m*/*z* 551.30 (**M7**), has a Mw 96 Da more than BA and 16 Da more than M5. Hence, M7 resulted most likely from both phase I and phase II metabolic reactions and consequently was assigned according to mass calculations to the monohydroxylated and sulfoconjugated BA metabolite, as depicted in Table 1. Moreover, the MS screening revealed the presence of a precursor ion detected at *m*/*z* 565.28 (**M8**), which has a Mw 110 Da more than BA and 30 Da more than M5. Thus, M8 is probably the product of both metabolic phases aswell, and was assigned according to mass calculations as the oxidated sulfoconjugated metabolite of BA; in this case the oxidation reaction converted the methyl group into carboxyl, as mentioned above. Moreover, the [M-H^+^]^−^ ion detected at *m*/*z* 567.30 (**M9**) which has a Mw 112 Da more than BA and 32 Da more than M5, presumably underwent both metabolic phases as well and thereupon was assigned according to mass calculations as the dihydroxylated sulfoconjugated metabolite of BA. M7, M8 and M9 were also described and associated with the in vivo metabolism of BA by Li et al. [23] following the intake of Ziziphi Spinosae semen extract in rats. Following these findings it must be emphasized that both BA and its phase I metabolites undergo sulfoconjugation during SKH1 female mice metabolism, data that are in line with previously published reports regarding in vivo triterpene metabolism [42] and, in particular, BA [23].

Next, the [M-H]^−^ identifiedat *m*/*z* 631.39 (**M10**) has a Mw 176 Da more than BA and was associated according to mass calculations to the glucuronide conjugate of BA. Glucuronidation is the most frequent phase II metabolic reaction and the most important detoxification pathway for both endogenous compounds and xenobiotics. Glucuronidation is catalyzed by UDP—glucuronosyltransferases (UGTs) that transfer UDP—glucuronic acid to nucleophilic atoms in the acceptor molecule [43]. Glucuronidation occurs at *O*–linked moieties such as hydroxy, phenolic or acyl and the resulting conjugates exhibit high hydrosolubility and consequently are easily eliminated via renal excretion or biliary excretion [38]. This type of conjugation also occurs both at the newly formed functional group during phase I reactions or directly with the parent ion that already possesses such a functional group. Hence, in the case of BA, most likely conjugation with GluA altered the already available hydroxyl group, as was reported before [23,44]. A large variety of phytocompounds undergo glucuronidation after in vivo administration in rodents, such as flavonoids [45], alkaloids [37] or triterpenes such as ursolic acid [44], betulinic acid [23] and betulin [46], thus enabling their excretion.

Inspection of the MS screening spectrum also indicated the formation of the [M-H]^−^ precursor ion at *m*/*z* = 647.38 (**M11**), which has a Mw 192 Da more than BA and 16 Da more than M10. Hence, according to the mass calculations, M11 is most likely the monohydroxylated glucuronoconjugate of BA. In a similar manner, the [M-H]^−^ ion detected at *m*/*z* 661.37 (**M12**) has a Mw 206 Da more than BA and 30 Da more than M10. Upon mass calculation, M12 has the features of being the oxidated glucuronoconjugated metabolite of BA, where the oxidation reaction altered the methyl group into carboxyl. Last, the [M-H]^−^ ion detected at *m*/*z* 663.37 (**M13**) has a Mw 208 Da more than BA and 32 Da more than M10 and thus was considered upon calculation as the dihydroxylated, glucuronoconjugated metabolite of BA. M10, M11 and M12 compounds associated with BA metabolism were also described by Li et al. [23], following the metabolomic profiling in rats subsequent to administration of traditional Chinese medicine remedies. As in the case of sulfoconjugates, it seems that both BA and its phase I metabolites undergo glucuronidation, as reported before for these types of molecules [42,47].

### 2.2. Structural Analysis of BA and BA Metabolites by Negative Ion Mode ESI Orbitratp MS/MS in Plasma Samples

The deprotonated ion [M-H]^−^ of BA and all the precursor ions related to BA metabolism identified in plasma samples were isolated and submitted to structural detailed investigation by fragmentation using CID MS/MSunder variable collision energies within 0–35 eV. The fragmentation features of BA and its metabolites consisted in neutral losses of H_2_O (−18 Da), CO_2_ (−44 Da), HCOOH (−46 Da), C_3_H_4_ (−40 Da), O (−16 Da), CH_2_ (−14 Da) and ring cleavage fragment ions, as already reported for triterpenes and triterpenic acids [22,32,40,43,47,48], but also for flavonoids [49] and other plant-derived glycosides [50].

#### 2.2.1. Structural Analysis of BA Isolated from Plasma Samples

**Figure 2:**
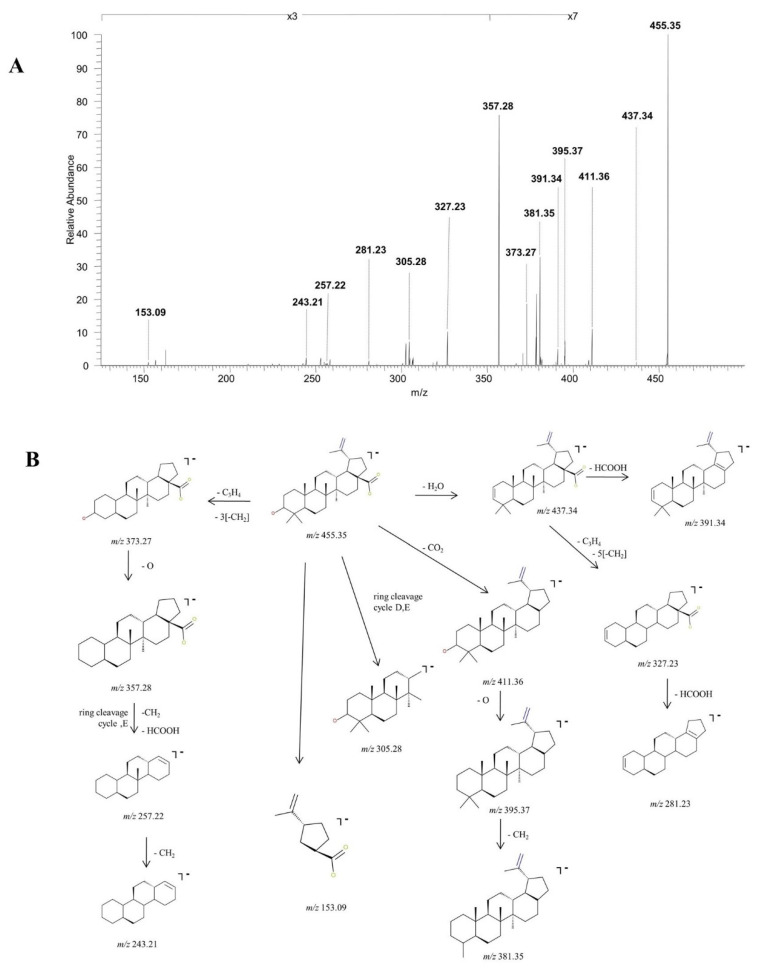
Structural characterization of betulinic acid (BA) (3β, hydroxy-lup-20(29)-en-28-oic acid). (**A**) ESI CID MS/MS spectra of the [M-H]^−^ ion detected at *m*/*z* 455.35 in the negative ion mode: spray voltage 0.70 kV, capillary temperature 275 °C and collision energies within 0–35 eV. (**B**) Proposed fragmentation pathway.

BA, identified as [M-H]^−^ at *m*/*z* 455.35, was isolated and submitted to structural analysis by CID MS/MS. The spectrum generated under these analytical conditions in depicted in Figure 2A and the fragmentation pathway of BA in Figure 2B. Inspection of the fragmentation spectrum indicates the formation of a high number of fragment ions diagnostic for the analyzed structure. The fragment ion identified at *m*/*z* 437.34 (−18 Da) corresponds to the neutral loss of water, under the form of [M-H^+^]^−^ H_2_O. The latter undergoes further fragmentation generating a fragment with 46 Da less, corresponding to the loss of HCOOH at *m*/*z* 391.34 and a fragment at *m*/*z* 327.23 by the cleavage of isopropenyl and five methylene substituents. The latter could further form a fragment at *m*/*z* 281.23 (−46 Da) by the elimination of HCOOH. Another sequencing pathway for BA is by the cleavage of CO_2_, yielding a fragment at *m*/*z* 411.36 (−44 Da). The decarboxylated form of BA undergoes several sequencing stages more generating a fragment ion at *m*/*z* 395.37 (−16 Da), corresponding to the loss of O, which in turn generates a fragment at *m*/*z* 381.35 (−14 Da), by the loss of CH_2_. Moreover, BA could also form the fragment at *m*/*z* 373.27 (−82 Da) by the cleavage of C_3_H_4_ and three CH_2_ substituents. The latter could yield a diagnostic fragment at *m*/*z* 357.28 (−16 Da) corresponding to the loss of O, that in turn could form *m*/*z* 257.22 by the loss of HCOOH, CH_2_ and ring cleavage of cycle E and subsequently *m*/*z* 243.21 by the loss of another CH_2_. Further, under these analytical conditions, *m*/*z* 305.28 and *m*/*z* 153.09, another two ring cleavage ions were identified.

#### 2.2.2. Structural Analysis of Phase I Metabolites

**Figure 3:**
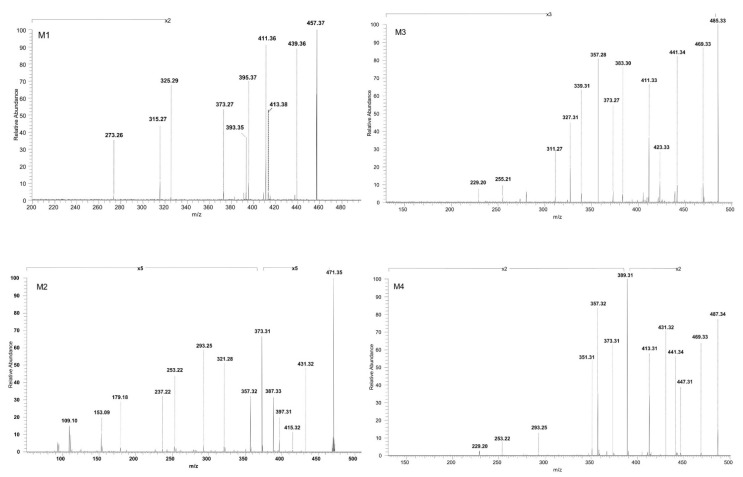
ESI CID MS/MS spectra in the negative ion mode of phase I metabolites detected as [M-H]^−^ ions: M1 (*m*/*z* 457.37), M2 (*m*/*z* 471.35), M3 (*m*/*z* 485.33) and M4 (*m*/*z* 487.34); spray voltage 0.70 kV, capillary temperature 275 °C and collision energies within 0–35 eV.

**Figure 4:**
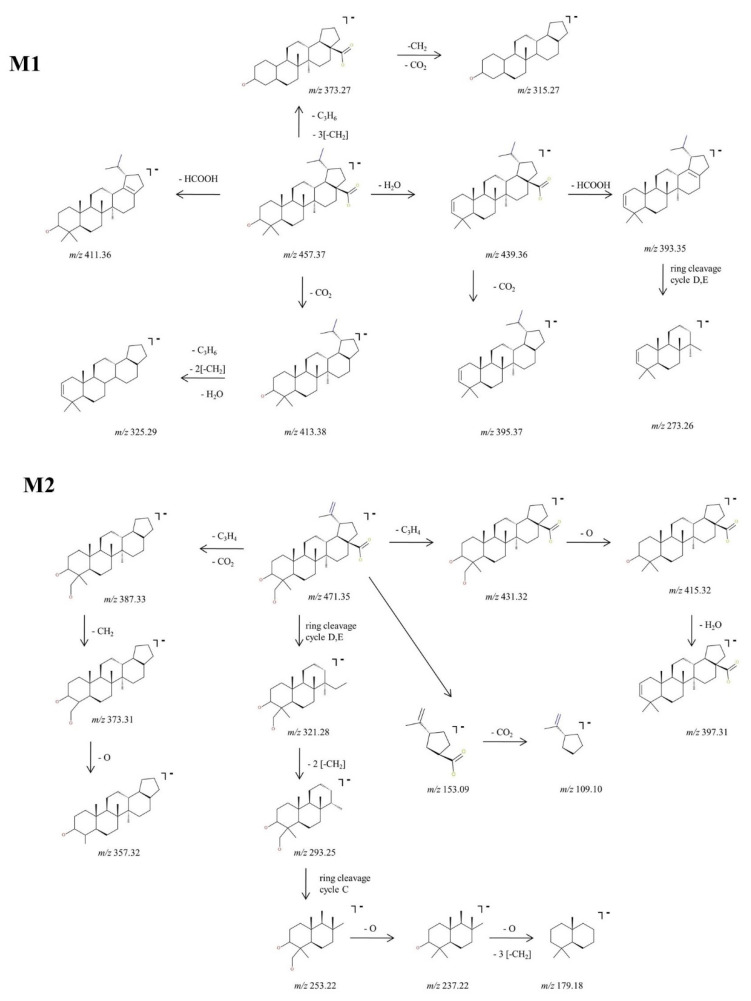
Proposed fragmentation pathway for phase I metabolites: M1–M2.

**Figure 5:**
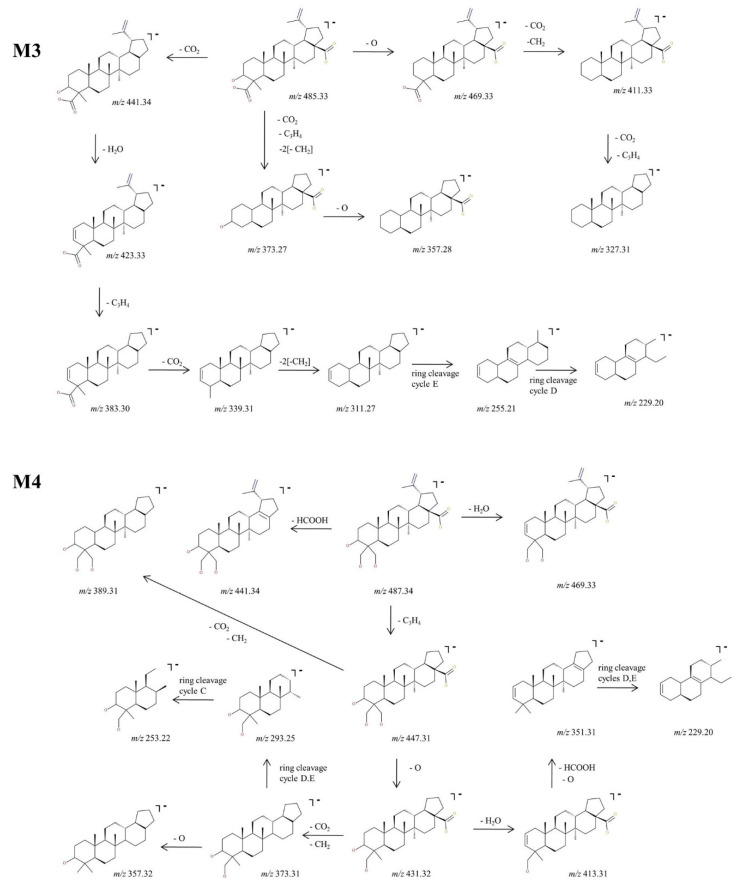
Proposed fragmentation pathway for phase I metabolites: M3–M4.

The experimental sequencing conditions by CID MS/MS enabled the isolation and structural investigation of the precursor ions related to phase I metabolism of BA resulting from monohydroxylation, dihydroxylation, oxidation and hydrogenation. ESI CID MS/MS spectra are depicted in Figure 3 and the proposed fragmentation pathway in Figure 4 and Figure 5.

M1, identified as the [M-H]^−^ ion at *m*/*z* 457.37 and assigned according to the mass calculations to the hydrogenation product of BA, was isolated and submitted to CID MS/MS sequencing, The fragmentation MS/MS spectrum and fragmentation scheme are depicted in Figure 3 and Figure 4. The fragment ion identified at *m*/*z* 439.36 (−18 Da) corresponds to the neutral loss of water and in turn forms a fragment ion at *m*/*z* 393.35 (−46 Da) by the cleavage of HCOOH and a fragment at *m*/*z* 395.37 (−44 Da) by the loss of CO_2_, a fragmentation pathway similar to the one of BA. Furthermore, the parent ion could yield two more fragment ions: (i) *m*/*z* 413.38 (−44 Da) by the loss of CO_2,_ which in turn yields to *m*/*z* 325.29 (−88 Da) by the concurrent loss of H_2_O, isopropyl and two CH_2_ substituents, and (ii) *m*/*z* 411.36 (−46 Da) by the loss of HCOOH. M1 could also yield the fragment ion at *m*/*z* 373.27 by the loss of isopropyl and three CH_2_ substituents_,_ which in turn generates the fragment at *m*/*z* 315.27 (−58 Da) by the loss of CO_2_ and CH_2_. Moreover, in the CID MS/MS spectrum, a ring cleavage fragment ion at *m*/*z* 273.26 was also identified.

M2, identified as [M-H]^−^ at *m*/*z* 471.35, was isolated and sequenced by CID MS/MS using a collision energy within 0–35 eV, parameters that enabled the formation of fragment ions suggestive for the monohydroxylated structure. The CID MS/MS spectrum of M2 is depicted in Figure 3 and the fragmentation scheme in Figure 4. The fragment ion identified at *m*/*z* 431.32 (−40 Da) corresponds to the loss of C_3_H_4_, and further forms *m*/*z* 415.32 (−16 Da) by the loss of O, which subsequently forms the fragment at *m*/*z* 397.31 (−18 Da) by neutral loss of H_2_O. Hence, the formation of the last two fragment ions are very suggestive for the proposed monohydroxylated metabolite structure. Furthermore, M2 could generate another fragment ion at *m*/*z* 387.33 (−84 Da) which corresponds to the loss of both C_3_H_4_ and CO_2_. The latter yields two more fragment ions at *m*/*z* 373.31 (−14 Da) and *m*/*z* 357.32 (−16 Da) by the consecutive loss of CH_2_ and O. Another fragmentation pathway for M2 occurred by a ring cleavage reaction that resulted in the loss of cycle E and opening of the cycle D, leading to the fragment ion identified at *m*/*z* 321.28 (−150 Da). The latter could further yield *m*/*z* 293.25 and *m*/*z* 253.22. by the consecutive loss of two CH_2_ followed by ring cleavage of cycle C. The last could form the fragment at *m*/*z* 237.22 (−16 Da) corresponding to the loss of O, followed by *m*/*z* 179.18 (−58 Da) by the concurrent elimination of another O and three CH_2_. All these data combined are indicative for the monohydroxylated structure. Not least, a small ring cleavage fragment was identified at *m*/*z* 153.09, as in the case of BA, together with its decarboxylated form at *m*/*z* = 109.10.

In addition, M3, identified as [M-H]^−^ ion at *m*/*z* 485.33 and assigned according to mass calculations to the oxidized metabolite of BA, was structurally characterized by tandem MS. The MS/MS spectrum reveals the formation of a substantial number of fragments that support the proposed dicarboxylated structure, as depicted in Figure 3 and Figure 5. The fragment ion identified at *m*/*z* 441.34 (−44 Da) corresponds to the loss of CO_2_, which in turn could form the fragment at *m*/*z* 423.33 (−18 Da) by the neutral loss of H_2_O. Most probably the latter could generate the fragment at *m*/*z* 383.30 (−40 Da) by the loss of C_3_H_4_ and further could form the fragment at *m*/*z* 339.31 (−44 Da) by the loss of the CO_2_ originating from the second available carboxyl group. In the MS/MS spectrum, the fragments at *m*/*z* 311.27, 255.21 and 229.20 that may derive from 339.23 were also identified, formed by the consecutive loss of CH_2_ groups and ring cleavage reactions, as proposed in the fragmentation scheme depicted in Figure 5. Another fragmentation pathway for the parent ion is associated with the fragment identified at *m*/*z* 469.33 (−16 Da) corresponding to the loss of O. The latter could generate the fragment at 411.33 (−58 Da) by the simultaneous loss of CO_2_ and CH_2_ groups, which in turn could further sequence with the formation of the fragment at *m*/*z* 327.31 (−84 Da) by the concurrent loss of CO_2_, originating from the second available carboxyl and C_3_H_4_ groups. Not least, the parent ion could undergo two consecutive fragmentation reactions, with the formation of two fragments at *m*/*z* 373.27 and 357.28, by the simultaneous loss of CO_2_, C_3_H_4_, two CH_2_ and the loss of O, respectively.

Next, M4, identified as the [M-H]^−^ ion at *m*/*z* 487.34 assigned according to mass calculation to the dihydroxylated metabolic product of BA, was structurally investigated. The MS/MS spectra (Figure 3) and proposed fragmentation pathway (Figure 5) exhibits the formation of a fair number of fragment ions that document the dihydroxylated structure. Hence, the parent ion could yield the following fragment ions: (i) *m*/*z* 469.33 (−18 Da), which corresponds to the neutral loss of a H_2_O; (ii) *m*/*z* 441.34 (−46 Da), which corresponds to the removal of HCOOH; and (iii) *m*/*z* 447.31 (−40 Da) by the elimination of C_3_H_4_. The latter could produce the fragment at *m*/*z* 389.31 (−58 Da) by the removal of CO_2_ and CH_2_ and also the fragment at *m*/*z* 431.32 (−16 Da) by the loss of O. The latter eliminates one molecule of H_2_O, generating the fragment at *m*/*z* 413.31 (−18 Da), which in turn could form the fragment ion at *m*/*z* 351.31 (−62 Da) by the concurrent removal of HCOOH and O. In addition, *m*/*z* 431.32 could produce the fragment at *m*/*z* 373.31 (−58 Da) by the elimination of CO_2_ and CH_2_, which in turn could form the fragment at *m*/*z* 357.32 by the removal of O. The sequencing parameter enabled the formation of a number of ring cleavage fragment ions such as *m*/*z* 229.20, which could derive from *m*/*z* 351.31, and *m*/*z* 293.25 and 253.22, originating from *m*/*z* 373.31 (Figure 5).

#### 2.2.3. Structural Analysis of Phase II Metabolites

**Figure 6:**
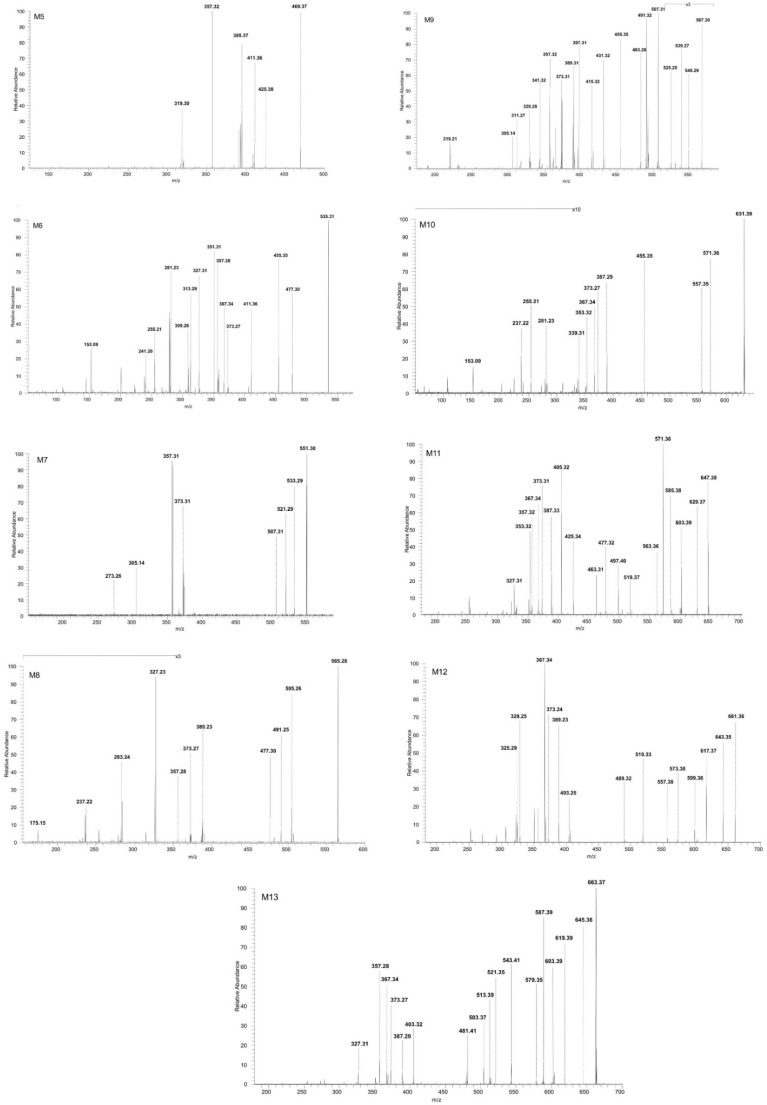
ESI CID MS/MS spectra in the negative ion mode of phase II metabolites detected as [M-H]^−^ ions: M5 (*m*/*z* 469.37), M6 (*m*/*z* 535.31), M7 (*m*/*z* 551.30), M8 (*m*/*z* 565.28), M9 (*m*/*z* 567.30), M10 (*m*/*z* 631.39), M11 (*m*/*z* 647.38), M12 (*m*/*z* 661.36) and M13 (*m*/*z* 663.37); spray voltage 0.70 kV, capillary temperature 275 °C and collision energies within 0–35 eV.

**Figure 7:**
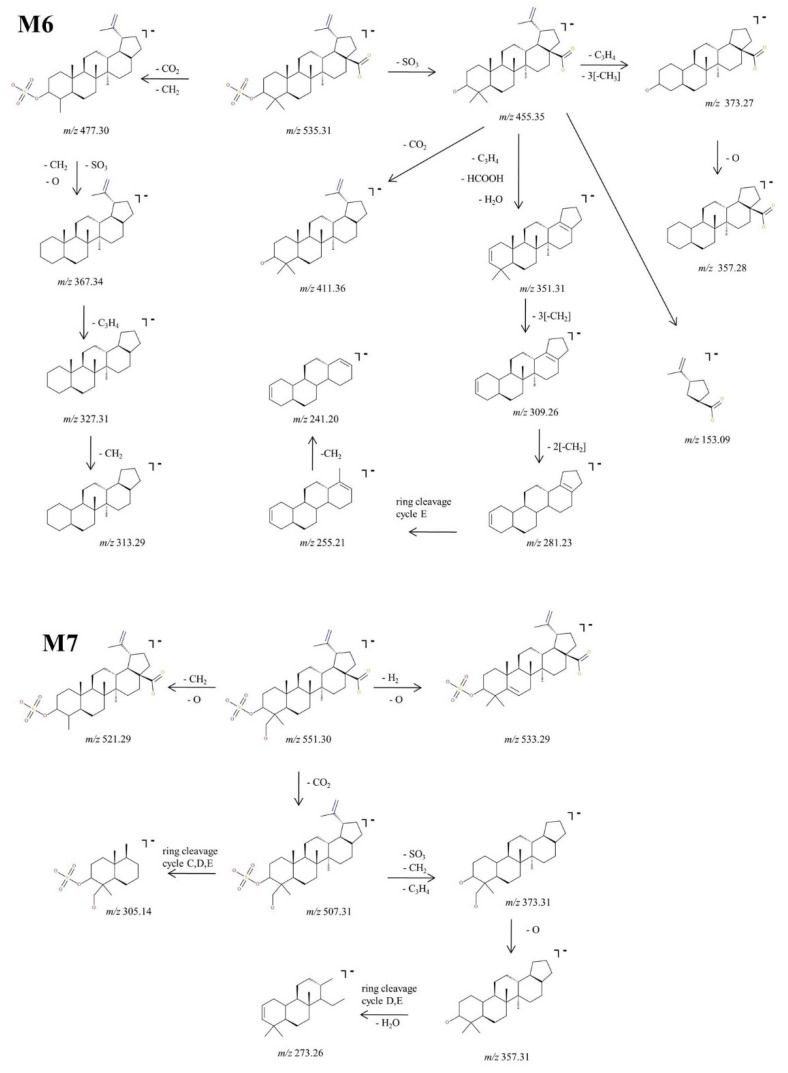
Proposed fragmentation pathway for phase II metabolites: M6–M7.

**Figure 8:**
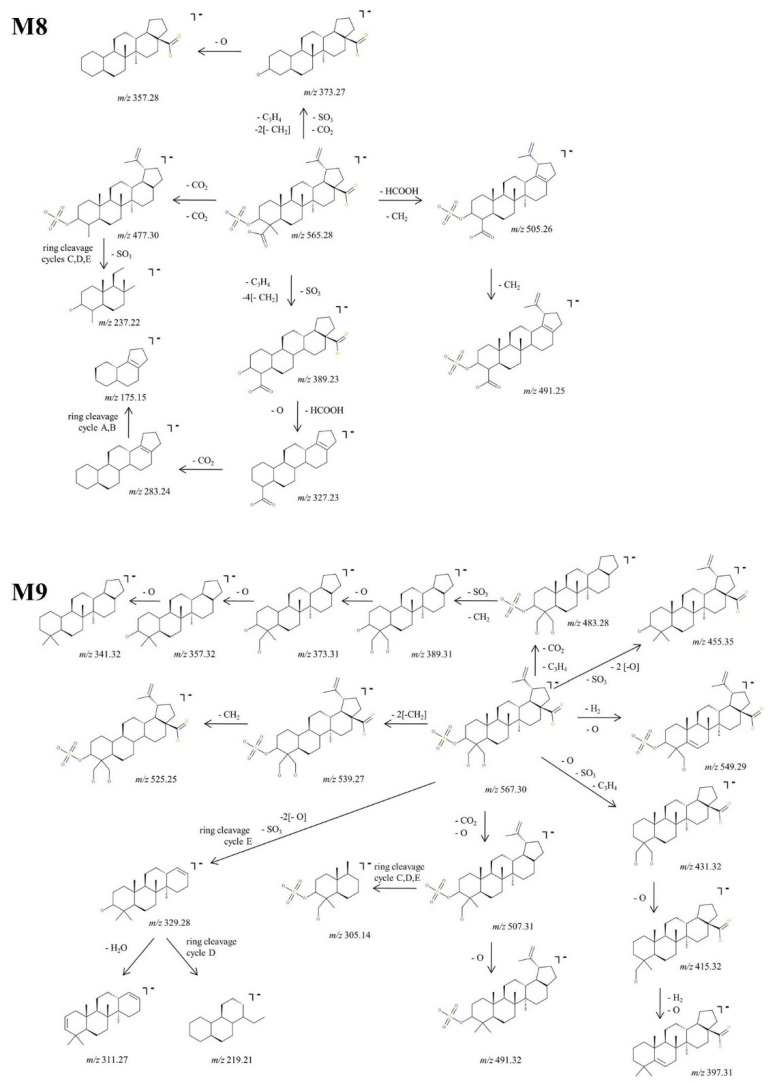
Proposed fragmentation pathway for phase II metabolites: M8–M9.

**Figure 9:**
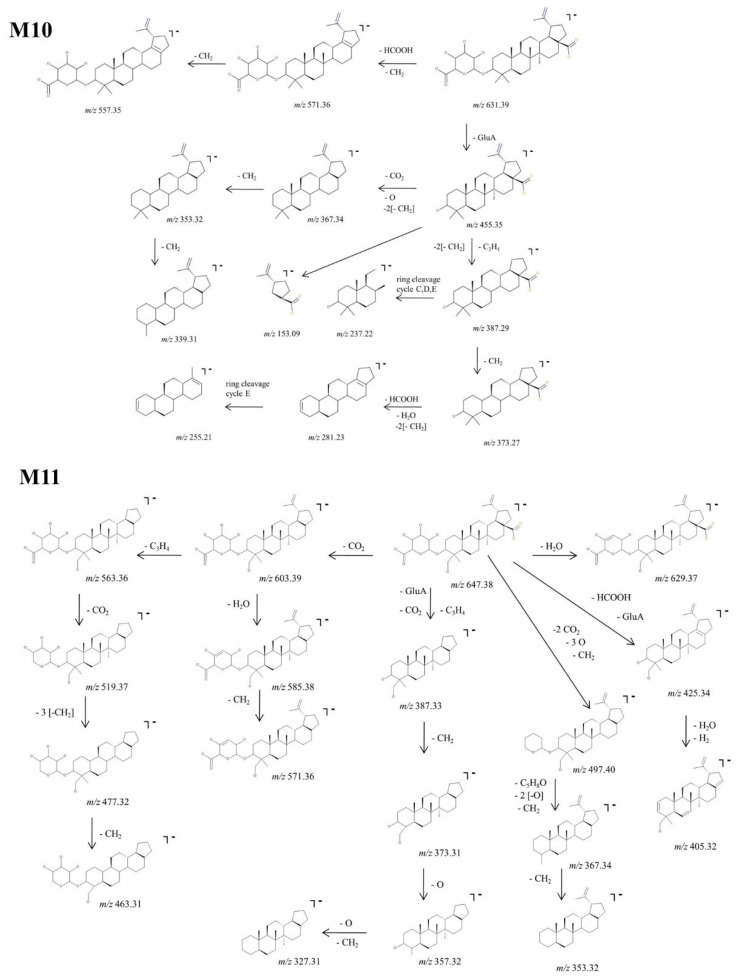
Proposed fragmentation pathway for phase II metabolites: M10–M11.

**Figure 10:**
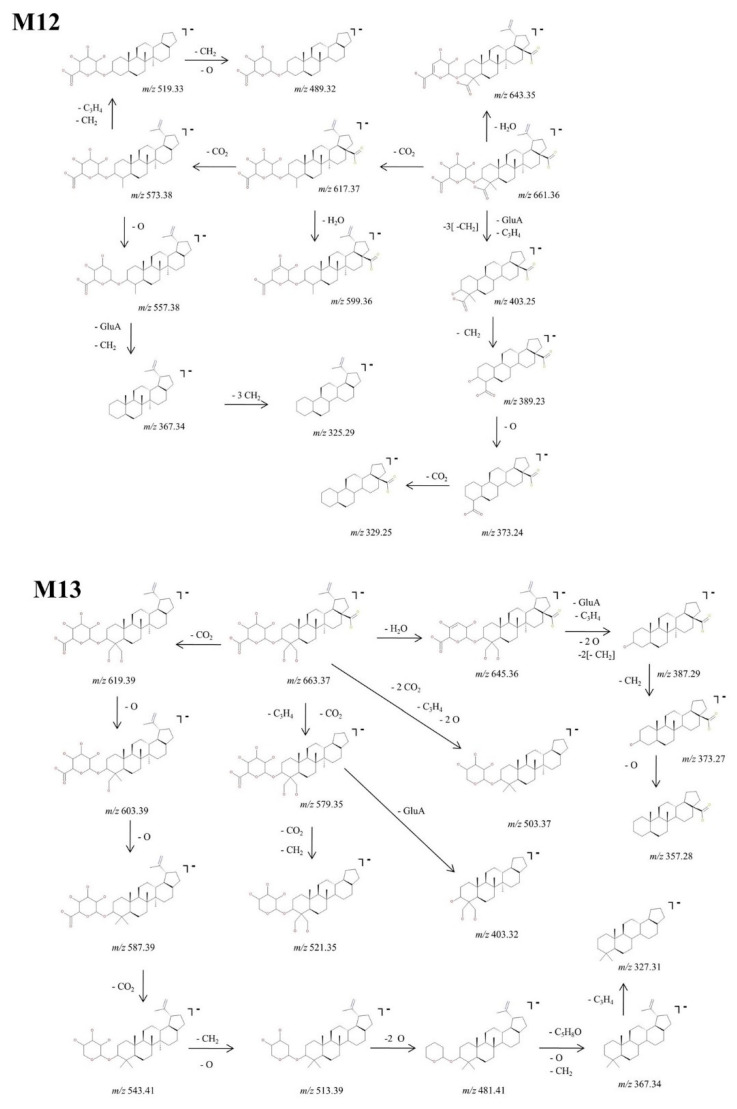
Proposed fragmentation pathway for phase II metabolites: M12–M13.

The precursor ions related to phase II metabolism of BA resulting from sulfation, glucuronidation and methylation were isolated and successfully sequenced by CID MS/MS. ESI CID MS/MS spectra are depicted in Figure 6 and the proposed fragmentation pathways in Figure 7, Figure 8, Figure 9 and Figure 10. M5 identified as the precursor ion [M-H]^−^ at *m*/*z* 469.37, assigned according to the mass calculations to the methylated metabolite of BA, was submitted to sequencing by CID MS/MS. The MS/MS spectra of M5 is depicted in Figure 6. The fragment ion at *m*/*z* 425.38 (−44 Da) corresponds to the loss of CO_2_. The latter could form a fragment ion at *m*/*z* 319.30(−106 Da) by the cleavage of cycle E and opening of cycle D and a fragment at *m*/*z* 411.36 (−14 Da) by the loss of CH_2_. In turn, *m*/*z* 411.36 could yield *m*/*z* 395.37 (−16 Da) by the elimination of O and *m*/*z* 357.32 by the cleavage of C_3_H_4_ and CH_2_.

##### Structural Analysis of Sulphated Metabolites

M6, identified as the precursor ion [M-H^+^]^−^ at *m*/*z* 535.31, was assigned according to the mass calculations to the sulfoconjugate metabolite of BA and was submitted to MS/MS experiments. Inspection of the MS/MS spectrum reveals the formation of a high number of diagnostic fragment ions that document the proposed structure. The fragmentation spectrum and proposed fragmentation pathway are depicted in Figure 6 and Figure 7. The fragment ion at *m*/*z* 455.35 is 80 Da less than the parent ion, a typical fragment for the sequencing of sulfoconjugates, which corresponds to the cleavage of SO_3_ [51] and most importantly brings solid evidence regarding the proposed sulfoconjugated structure. Further, *m*/*z* 455.35 could generate: (i) the fragment at *m*/*z* 411.36 (−44 Da) which corresponds to the removal of CO_2_, as presented in the fragmentation scheme of BA; (ii) the fragment at *m*/*z* 351.31 (−104 Da) by the simultaneous loss of HCOOH, C_3_H_4_ and H_2_O, which in turn could produce the fragment at *m*/*z* 309.26 (−42 Da) by the removal of three CH_2_ groups, which generates the fragment at *m*/*z* 281.23 (−28 Da) by the elimination of other two CH_2_ groups, lastly forming two consecutive ring cleavage ions at *m*/*z* 255.21 and 241.20 corresponding to the elimination of cycle E; (iii) *m*/*z* 373.27 (−82 Da), corresponding to the simultaneous cleavage of C_3_H_4_ and three CH_2_ groups, which in turn could produce *m*/*z* 357.28 (−16 Da), corresponding to the additional removal of O, as already presented in the fragmentation scheme of BA. Moreover, the MS/MS spectra of M5 exhibit a fragment ion at *m*/*z* 477.30 (−58 Da) which could be produced by the concurrent elimination of CO_2_ and CH_2_, that in turn could generate the fragment at *m*/*z* 367.34 (−110 Da) by the simultaneous removal of SO_3_, O and CH_2_. The latter could form two consecutive fragments at *m*/*z* 327.31 (−40 Da) and 313.29 (−14 Da) by the loss of C_3_H_4_ and CH_2_,respectively. Nonetheless, a small fragment at *m*/*z* 153.09 was also identified, which corresponds to cycle E and its substituents, as already observed in the MS/MS spectrum of BA or M2.

Next, M7 was identified as the precursor ion [M-H]^−^ at *m*/*z* 551.30, which according to the mass calculations corresponds to the monohydroxylated sulfoconjugated metabolite of BA, and was structurally investigated during MS/MS experiments. The fragmentation spectrum and proposed fragmentation pathway are depicted in Figure 6 and Figure 7. Inspection of the MS/MS spectra reveals the formation of the fragment ion at *m*/*z* 507.31 (−44 Da), which corresponds to the elimination of CO_2_. The latter could form the following fragments: (i) *m*/*z* 305.14 (−202 Da), which correlates to a ring cleavage fragment ion of rings C, D, E, bearing the sulphate; (ii) *m*/*z* 373.31 (−134 Da) by the simultaneous loss of SO_3_, C_3_H_4_ and CH_2_ groups. The latter could produce the fragment at *m*/*z* 357.31 (−16 Da) by the removal of O and last, a ring cleavage ion of cycles D, E concurrent with loss of H_2_O at *m*/*z* 273.27. Two other fragment ions were identified in the MS/MS spectra of the parent ion: *m*/*z* 533.29 which corresponded to the loss of O and desaturation, and *m*/*z* 521.29 (−30 Da), which correlated to the concurrent removal of CH_2_ and O.

M8, identified as the [M-H]^−^ ion *m*/*z* 565.28, was assigned according to mass calculations as the oxidated sulfconjugated metabolite of BA and was submitted to MS/MS experiments. The MS/MS spectrum and proposed fragmentation pathway depicted in Figure 6 and Figure 8 exhibit the formation of a fair number of fragment ions that document the proposed structure. M8 could generate four fragment ions: (i) *m*/*z* 477.30 (−88 Da) by the loss of 2 CO_2_ derived from the available two carboxyl groups, followed by a ring cleavage fragment concurrent with the loss of SO_3_ at *m*/*z* 237.22; (ii) *m*/*z* 389.23 (−176 Da) by the concurrent cleavage of SO_3_, C_3_H_4_ and four CH_2_ groups, which it turn could form *m*/*z* 327.23 (−62 Da) by the cleavage of HCOOH and O, *m*/*z* 283.24 (−44 Da) by the loss of CO_2_ and finally *m*/*z* 175.15 by the cleavage of cycles A and B; (iii) *m*/*z* 505.26 (−60 Da) by the cleavage of HCOOH and CH_2_ groups followed by *m*/*z* 491.25 (−14 Da) resulting from the loss of another CH_2_; (iv) *m*/*z* 373.27 (−192 Da) by the concurrent elimination of SO_3_, CO_2_, C_3_H_4_ and two CH_2_ groups, which in turn could generate both *m*/*z* 357.28 by the loss of O.

M9, identified as the [M-H]^−^ ion at *m*/*z* 567.30, was assigned according to the mass calculations as the dihydroxylated sulfoconjugated BA metabolite and was isolated and submitted to detailed structural analysis by MS/MS experiments. Inspection of the fragmentation spectra (Figure 6) indicates the formation of a large number of fragment ions that document the metabolite structure, as depicted in the proposed fragmentation pathway (Figure 8). Thus, one of the most important fragment ions identified in the fragmentation spectra is *m*/*z* 455.35 (−112 Da), corresponding to BA resulting from the concurrent removal of SO_3_ and two O and providing solid evidence for the structure of the proposed metabolic product. Additionally, M9 could generate the following fragments: (1) *m*/*z* 431.32 (−136 Da) by the simultaneous loss of SO_3_, O and C_3_H_4_ groups, that in turn forms the fragment at *m*/*z* 415.32 (−16 Da) by the elimination of O and consecutively *m*/*z* 397.31 by the loss of O and desaturation; (2) *m*/*z* 507.30 (−60 Da) by the removal of CO_2_ and O groups, which in turn could produce the fragment at *m*/*z* 491.32 (−16 Da) by the elimination of O and a ring cleavage fragment at *m*/*z* 305.14, which bears SO_3_; (3) *m*/*z* 539.27 (−28 Da) by the removal of two CH_2_ groups and consecutively *m*/*z* 525.25 (−14 Da) by the loss of another CH_2_; (4) *m*/*z* 483.28 (−84 Da) by the concomitant loss of CO_2_ and C_3_H_4_ groups, which in turn could produce the fragment at *m*/*z* 389.31 (−94 Da) by the simultaneous cleavage of SO_3_ and CH_2_; the latter could form a series of three fragments at *m*/*z* 373.31, *m*/*z* 357.32 and *m*/*z* 341.32 by the successive loss of O, originating from the three available hydroxyl substituents, hence reinforcing the proposed dihydroxylated sulfoconjugated structure; (5) *m*/*z* 549.31, corresponding to the loss of O and desaturation; (6) *m*/*z* 329.28, a ring cleavage ion that could also derive from the parent ion, which in turn could produce *m*/*z* 311.27 by the loss of H_2_O and *m*/*z* 219. 21, a ring cleavage ion of cycle D.

##### Structural Analysis of Glucuronated Metabolites

M10, identified as the precursor ion [M-H]^−^ at *m*/*z* 631.39, was assigned according to the mass calculations as the glucuronoconjugate of BA and was submitted to structural investigation by CID MS/MS. The fragmentation spectra (Figure 6) indicates the formation of fragment ions diagnostic for the glucuronoconjugated structure, as depicted in the proposed fragmentation pathway (Figure 9). M10 could produce the following fragment ions: (i) *m*/*z* 571.36 (−60 Da) by the cleavage of HCOOH and CH_2_ groups, that in turn forms *m*/*z* 557.35 (−14 Da) by the loss of another CH_2_; (ii) *m*/*z* 455.35 (−176 Da) by the cleavage of GluA from the parent ion, a typical fragmentation pattern for glucuronoconjugates [52], which provides solid evidence for the glucuronidation of BA. The latter could generate the following: (i) *m*/*z* 367.34 (−88 Da) by the loss of CO_2_, two CH_2_ and O, that in turn could form *m*/*z* 353.32 by the loss of CH_2_ and consecutively *m*/*z* 339.31 by the loss of another CH_2;_; (ii) *m*/*z* 387.29 (−68 Da) by the elimination of C_3_H_4_ and two CH_2_ groups. The latter could generate *m*/*z* 373.27 (−14 Da) by the removal of CH_2_, which in turn forms *m*/*z* 281.23 (−92 Da) by the concurrent loss of HCOOH, H_2_O and two CH_2_ groups, and *m*/*z* 255.21, a ring cleavage of cycle E. Nonetheless, the MS/MS spectra exhibited also *m*/*z* 237.22, a ring cleavage ion probably originating from *m*/*z* 387.29 and *m*/*z* 153.09, a ring cleavage ion already observed in the spectrum of BA or M6.

Next, M11, identified as the [M-H]^−^ ion at *m*/*z* 647.38, was assigned according to mass calculations as the monohydroxylatedglucuronoconjugated metabolite of BA and was submitted to structural experiments by CID MS/MS. Inspection of the MS/MS spectra (Figure 6) and proposed fragmentation pathway (Figure 9) indicates the formation of a fair number of fragment ions that support the proposed structure. Hence, M11 could generate *m*/*z* 603.39 (−44 Da) by the neutral loss of CO_2,_ which in turn could undergo further sequencing with the formation of two fragments: (i) *m*/*z* 563.36 (−40 Da) by the loss of C_3_H_4_, which in turn generates three more fragments at *m*/*z* 519.37 (−44 Da), 477.32 (−42 Da) and 463.31 (−14 Da) by the consecutive elimination of CO_2_, three CH_2_ and one more CH_2_, respectively; (ii) *m*/*z* 585.38 (−18 Da) by the neutral loss of H_2_O, which in turn forms *m*/*z* 571.36 (−14 Da) by the elimination of CH_2_. **M11** could generate four more fragment ions: (1) *m*/*z* 387.33 (−260 Da) by the concurrent cleavage of GluA, CO_2_ and C_3_H_4_, which further could form *m*/*z* 373.31 (−14 Da), 357.32 (−16 Da) and 327.31 (−30 Da) by the consecutive loss of O and CH_2_; (ii) *m*/*z* 497.40 (−150 Da) by the elimination of the available two CO_2,_ three CH_2_ and three O, which further produces *m*/*z* 367.34 and *m*/*z* 353.32; (iii) *m*/*z* 425.34 (−222 Da) by the cleavage of GluA and HCOOH, followed by dehydratation and desaturation, generating *m*/*z* 405.32; (iv) *m*/*z* 629.37 (−18 Da) by the neutral loss of H_2_O.

M12, identified as the [M-H]^−^ ion *m*/*z* 661.36, was assigned according to mass calculations as the oxidated glucuronoconjugated metabolite of BA. The fragmentation CID MS/MS spectrum and proposed fragmentation pathway are depicted in Figure 6 and Figure 10. M12 could form *m*/*z* 617.37 (−44 Da) by the loss of CO_2_ that in turn forms both *m*/*z* 599.36 (−18 Da) by the neutral loss of H_2_O and *m*/*z* 573.38 (−44 Da) by the cleavage of another CO_2_. The latter could form: (i) *m*/*z* 557.38 (−16 Da) by the loss of O, together with two fragments at *m*/*z* 367.34 and 325.90; (ii) *m*/*z* 519.33 (−54 Da) and 489.32 (−30 Da) by the consecutive loss of two CH_2_, C_3_H_4_ and O. Further, M12 could generate *m*/*z* 643.35 (−18 Da) by the neutral loss of H_2_O and 403.25 by the concurrent cleavage of GluA, C_3_H_4_ and three CH_2_ groups. The latter could form a series of fragments at *m*/*z* 389.23, 373.24 and 329.25 by the consecutive cleavage of CH_2_, O and CO_2_.

At last, M13, identified as the precursor ion [M-H]^−^ ion at *m*/*z* 663.37, was assigned according to mass calculations as the dihydroxylated glucuronoconjugated metabolite of BA and was submitted to structural experiments by CID MS/MS. The fragmentation spectrum and proposed fragmentation pathway are depicted in Figure 6 and Figure 10. M13 could yield the following fragment ions: (i) *m*/*z* 619.39 (−44 Da) by the loss of CO_2_, and subsequently *m*/*z* 603.39 (−16 Da) and 587.39 (−16 Da) by the consecutive loss of O. The latter could form *m*/*z* 543.41 (−44 Da) by the elimination of another CO_2_, followed by *m*/*z* 513.39 (−30 Da) by the cleavage of CH_2_ and O, which in turn forms *m*/*z* 481.41 (−32 Da) by the loss of O. The latter could also further generate *m*/*z* 367.34 by the cleavage of C_5_H_8_O, CH_2_ and O and consecutively *m*/*z* 327.31 by the additional loss of C_3_H_4_; (ii) *m*/*z* 645.36 (−18 Da) by loss of H_2_O, which in turn exhibits extensive fragmentation by the cleavage of GluA, C_3_H_4_, two O and two CH_2_ groups, generating *m*/*z* 387.29 (−258 Da). The latter could be fragmented further and form *m*/*z* 373.27 (−14 Da) and 357.28 (−16 Da) by the consecutive loss of CH_2_ and O, respectively; (iii) *m*/*z* 503.37 (−160 Da) by the elimination of two CO_2_, two O and C_3_H_4_ groups; and (iv) *m*/*z* 579.35 (−84 Da) by the cleavage of CO_2_, and C_3_H_4_. The latter could further generate *m*/*z* 403.32 (−176 Da) by the cleavage of GluA and *m*/*z* 521.35 (−58 Da) by the loss of CO_2_ and CH_2_, respectively.

### 2.3. Proposed Metabolic Pathway for BA in SKH1 Female Mice

**Figure 11:**
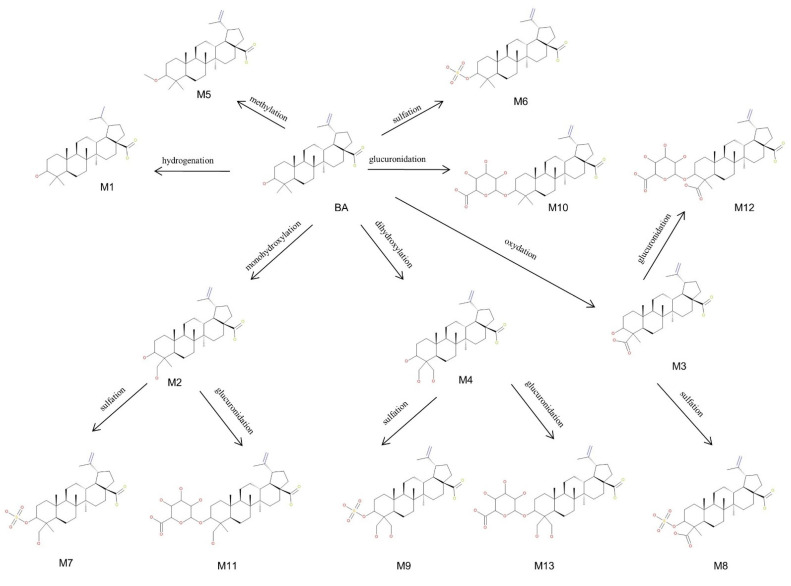
Proposed metabolic pathway for betulinic acid (BA) (3β, hydroxy-lup-20(29)-en-28-oic acid) in SKH1 female mice.

In the current research, we were able to identify a total of 13 metabolites of BA after intraperitoneal administration to SKH1 female mice, as depicted in Figure 11. Out of the total metabolites, four (M1–M4) were associated with phase I metabolism resulting from metabolic reactions such as monohydroxylation, dihydroxylation, oxidation and hydrogenation. During these reactions, the targets for metabolic alterations were the isopropenyl and methyl substituents of BA. In addition, nine (M5–M13) more metabolites, correlated with phase II metabolism, were identified. The main phase II metabolic reactions for BA were sulfation, glucuronidation and methylation, out of which: (i) three resulted directly from phase II metabolism (M5, M6, M10) and (ii) six resulted from sulfoconjugation and glucuronidation of phase I metabolites (M7–M9, M11–M13) yielded by monohydroxylation, dihydroxylation and oxidation reactions. The metabolic site for M5, M6, M10 was the only available hydroxyl substituent of BA, as described before [38]. Since BA metabolism exhibited both compounds resulting from direct conjugation with endogenous substrates and compounds conjugated with phase I metabolites, as disclosed in a previous study [23], we may assume that the metabolic target for the conjugation of M7–M9 and M11–M13 metabolites could also be the original hydroxyl substituent of BA.

Although during the CID MS/MS structural investigations, a lot of diagnostic fragments for the proposed structures were exhibited, more confirmation is needed for the exact metabolic sites and alterations. For a future perspective, the current research will be completed with multistage sequencing (MS^n^) of the metabolic products and NMR confirmation of their structure.

## 3. Materials and Methods

### 3.1. Reagents

#### 3.1.1. Chemicals

BA was purchased from Sigma-Aldrich (Taufkirchen, Germany). Acetone, methanol, flax-seed oil and egg phosphatidylcholine were purchased from Merck (Darmstadt, Germany) and used without further purification.

#### 3.1.2. BA Nanoemulsion

BA nanoemulsion was obtained by reproducing a previously reported technique [53]. Briefly, BA was prepared as an oil-in-water nanoemulsion by homogenizing flax-seed oil (the internal oil phase) with BA dissolved in chloroform. The aqueous phase containing dissolved egg phosphatidylcholine in deionized ultrapure water was added under constant stirring, followed by homogenizing cycles.

### 3.2. In Vivo Experiment

For the in vivo studies, SKH1 adult female mice (*n* = 4 mice, age: 20–24 weeks, weight: 25 ± 2 g) were acquired from Charles River Laboratory (Budapest, Hungary) and used. The animals were kept in the University animal facility in standard conditions, as follows: food and water ad libitum, a 12 h/12 h light-dark cycle, an ambient temperature of 22–24 °C, and humidity around 55%. The experimental procedures were performed in compliance with the European Directive 2010/63/EU, the AVMA Guidelines for the Euthanasia of Animals (2013 Edition) and the National Law 43/2014 regarding the protection of animals used for scientific purposes and were analyzed and approved by the University Research Ethics Committee. The following protocol was applied: each mouse was intraperitoneally injected with a dose of 40 mg/kg body weight BA nanoemulsion. Blood samples were collected after 2 h post administration by using the periorbital technique also known as orbital venous plexus bleeding, after a standardized protocol [54]. All the procedures were performed under anesthesia provided by isoflurane inhalation. At the end of the experiment, the mice were euthanized by anesthesia and cervical dislocation.

### 3.3. Plasma Samples

Blood was collected in sterile vials on ethylenediaminetetraacetic acid (EDTA), centrifuged for 10 min at 10,000 rpm and then plasma was collected and stored at −20 °C. All plasma samples were deproteinized with methanol by dissolving 1 part plasma with 3 parts methanol, respectively (*v*/*v*). The supernatant was collected and subjected to another round of purification with acetone by the same protocol. All sample solutions were homogenized with a WisdVM-10vortex mixer (Witeg Labortechnik, Wertheim, Germany) and centrifuged for 2 min at 10,000 rpm in a ThermoMicro CL17 microcentrifuge (Thermo Fisher Scientific, Massachusetts, MA, USA). The supernatant was collected and submitted to MS measurements.

### 3.4. Orbitrap Mass Spectrometry

The nanoESI MS experiments were conducted on a LTQ Orbitrap Velos Pro^TM^ mass spectrometer, from Thermo Fisher Scientific (Bremen, Germany), equipped with the offline nanoES source ES 259 (Thermo Fisher, Massachusetts, MA, USA). Ten microliters from each plasma sample was loaded into the borosilicate emitters ES380 (Proxeon, Roskilde, Denmark) and directly infused into the instrument through the offline nanoES source connected to the instrument using the Nanospray Flex Ion Source (Thermo Scientific, Massachusetts, MA, USA) at a spray current of 0.08 µA, obtained by applying a spray voltage of 0.70 kV, keeping the capillary temperature at 275 °C and the S-lens RF level at 60%. All mass spectra were subsequently screened in negative ion mode, which was previously demonstrated to be the best option for molecules containing carboxylic moiety [55], and detected under identical conditions, with no sheath, sweep or auxiliary gas, in an *m*/*z* range of 100 to 1000. Prior to experiments, the *m*/*z* scale was calibrated with external standard Pierce ESI Negative Ion Solution (Thermo Scientific, Massachusetts, MA, USA). The mass spectrometer was operated and controlled by the LTQ Tune Plus v2.7 build 1112 SP2 software (Thermo Scientific, Massachusetts, MA, USA) running under Windows 7, while the MS data acquisition and processes were achieved using Xcalibur 3.0.63 software (Thermo Scientific, Massachusetts, MA, USA). MS/MS experiments were carried out in the LTQ sector by CID with Orbitrap detection. Ion selection and fragmentation were performed manually, using variable collision energies within 0–35 eV. The precursor ions were selected within an isolation width of 2 *m*/*z* units.

## 4. Conclusions

The current study was focused on the screening and sequencing of BA metabolites by MS-based techniques, following in vivo administration. To this end, a nanoemulsion containing BA was prepared and administered intraperitoneally to SKH1 female mice. Plasma samples were collected post-treatment and were analyzed by HRMS. Screening experiments of BA and BA metabolites in plasma samples were conducted by ESI Orbitrap MS in the negative ion mode, while structural characterization was conducted by CID MS/MS. The study design combined with the applied analytical strategy enabled the identification of 13 phase I and phase II metabolites of BA in plasma samples. The main phase I metabolic reactions BA underwent were monohydroxylation, dihydroxylation, oxidation and hydrogenation, while phase II metabolism was represented by a conjugation reaction with sulfate or GluA and methylation. In addition, detailed structural information were collected by sequencing of the precursor ions by CID MS MS, data that enabled elucidation of the fragmentation pathway for BA and its plasma metabolites. The main fragmentation pattern for BA and its phase I metabolites revealed during MS/MS experiments consisted mainly in neutral losses of H_2_O, CO_2,_ HCOOH, C_3_H_4_, O and CH_2_ accompanied in some cases by ring cleavage fragmentation. In addition, phase II metabolites exhibited diagnostic fragments by the cleavage of sulfate and GluA. The whole set of information obtained during the detailed structural investigation of BA metabolites contributes to framing a solid database of BA’s metabolic pathway and provides in-depth knowledge of their CID MS/MS sequencing behavior. The obtained results enrich and complete BA’s ADME parameters, in particular its metabolic profile, mandatory for all molecules that are considered to be developed as medicinal drugs. Moreover, some of the identified metabolites during the present study may be active metabolites, which are most likely more polar than BA and possess superior bioavailability. Hence, this work represents a foundation for developing BA derivatives that share its biological and pharmacological activities but have the credentials to display a superior pharmacokinetic profile, suitable for cancer treatment in humans.

## Figures and Tables

**Table 1:** Proposed phase I and phase II metabolic products of betulinic acid (BA) (3β, hydroxy-lup-20(29)-en-28-oic acid).

Metabolite	Molecular Formula	*m*/*z*	Molecular Ion	Metabolic Reaction	Molecular Alteration	Mass Change
M 1	C_30_H_50_O_3_	457.37	[M-H^+^]^−^	-hydrogenation	+2H	+2 Da
M 2	C_30_H_48_O_4_	471.35	[M-H^+^]^−^	-monohydroxylation	+O	+16 Da
M 3	C_30_H_46_O_5_	485.33	[M-H^+^]^−^	-oxydation	+2O −2H	+30 Da
M 4	C_30_H_48_O_5_	487.34	[M-H^+^]^−^	-dihydroxylation	+2O	+32 Da
M 5	C_31_H_50_O_3_	469.37	[M-H^+^]^−^	-methylation	+CH_2_	+14 Da
M 6	C_30_H_48_O_6_S	535.31	[M-H^+^]^−^	-sulfoconjugation	+SO_3_	+80 Da
M 7	C_30_H_48_O_7_S	551.30	[M-H^+^]^−^	-hydroxylation -sulfoconjugation	+O +SO_3_	+96 Da
M 8	C_30_H_46_O_8_S	565.28	[M-H^+^]^−^	-oxidation -sulfoconjugation	+2O −2H +SO_3_	+110 Da
M 9	C_30_H_48_O_8_S	567.30	[M-H^+^]^−^	-dihydroxylation -sulfoconjugation	+2O +SO_3_	+112 Da
M 10	C_36_H_56_O_9_	631.39	[M-H^+^]^−^	-glucuronidation	+GluA	+176 Da
M 11	C_36_H_56_O_10_	647.38	[M-H^+^]^−^	-hydroxylation -glucuronidation	+O +GluA	+192 Da
M 12	C_36_H_54_O_11_	661.36	[M-H^+^]^−^	-oxydation -glucuronidation	+2O −2H +GluA	+206 Da
M 13	C_36_H_56_O_11_	663.37	[M-H^+^]^−^	-dihydroxylation -glucuronidation	+2O +GluA	+208 Da

## Data Availability

Not applicable.
